# Evaluating 3D Morphometrics for Assessing Micrognathia in Neonates with Severe Pierre Robin Sequence: A Case-Control Study

**DOI:** 10.1177/10556656261431485

**Published:** 2026-03-27

**Authors:** Sahejbir S. Bhatia, Paul W. Hung, Alexander E. Velazquez, Kayla Prezelski, Lucas M. Harrison, Timothy Booth, Rami R. Hallac, Alex A. Kane, Paymon Sanati-Mehrizy

**Affiliations:** 1 Department of Plastic Surgery, University of Texas Southwestern Medical Center, Dallas, TX, USA; 2 Department of Radiology, University of Texas Southwestern Medical Center, Dallas, TX, USA; 3 Analytical Imaging and Modeling Center, Children's Health Medical Center, Dallas, TX, USA

**Keywords:** Pierre Robin sequence, micrognathia, distraction, osteogenesis, cephalometry, anthropometry

## Abstract

**Objective:**

To compare 3D mandibular morphometrics of neonates with Pierre Robin sequence (PRS) to age-matched controls, establish normative data, and better understand anatomic differences.

**Design:**

Retrospective case-control study.

**Setting:**

Tertiary pediatric medical center.

**Patients:**

Twenty-five neonates with non-syndromic PRS (mean age 15 days) with computed tomography (CT) scans of the head and mandibular distraction osteogenesis (MDO) and 25 age-matched controls (mean age 14 days) with CT scans of the head.

**Interventions:**

None.

**Main Outcome Measures:**

Standardized morphometric measurements, including linear distances, angles, and ratios, were generated from digital 3D reconstructions of mandibular anatomy.

**Results:**

Asymmetry in PRS body length was significantly higher than in control mandibles (*p* = .006). PRS measurements larger than control measurements included intercondylar distance (*p* = .044), intergonial distance (*p* = .008), pogonion-interdental distance (*p* = .015), ramus height (*p* = .001), and ramus height to body length ratio (*p* < .001). PRS measurements smaller than control measurements included intercondylar angle (*p* < .001), body length (*p* < .001), and effective mandibular length (*p* < .001).

**Conclusions:**

In a cohort of neonates with PRS severe enough to warrant MDO, findings indicate that micrognathia is constituted by a wide and antero-posteriorly short mandible for its height. Except for body length, PRS and control mandibles are similarly asymmetrical in other dimensions. These findings may contextualize structural abnormalities contributing to glossoptosis and airway obstruction in PRS, with implications for surgical management, although decision-making remains primarily guided by clinical severity of airway obstruction.

## Background

Pierre Robin sequence (PRS) is a congenital condition characterized by a triad of features: micrognathia, glossoptosis, and airway obstruction.^1–3^ This condition often presents with additional complications, such as cleft palate, and is associated with significant challenges in neonatal care due to respiratory and feeding difficulties.^3,4^ While micrognathia and glossoptosis are structural hallmarks of PRS, the indication for intervention and choice of therapy is ultimately determined by the severity and pattern of upper airway obstruction in addition to the mandibular morphology.

Micrognathia, a key feature of PRS, plays a critical role in the pathophysiology of the sequence. The reduced mandibular size limits the oral cavity capacity, causing posterior tongue displacement, which in turn can lead to airway obstruction.^1–4^ Recent quantitative studies have characterized the distinct mandibular morphology in neonates with PRS compared to controls. Infants with PRS demonstrate shorter mandibular sagittal lengths, shorter ramus and body lengths, steeper gonial angles, and narrower symphyseal angles compared to age-matched controls without the condition (Figure 1).^5–8^ Additionally, prior 3D morphometric studies have shown that isolated and non-isolated PRS mandibles have shorter condylar necks, shorter mandibular bodies, and less pronounced, more rounded symphyseal projection.^7^ While these characteristics of micrognathia in PRS have been explored, the presence of mandibular asymmetry remains an important area requiring further investigation, as asymmetric anomalies of the mandible in PRS can vary based on the specific diagnosis.^1–8^ Understanding patterns of asymmetry, if present, could have implications for surgical planning, particularly in determining optimal osteotomy design and distraction vectors for mandibular distraction osteogenesis (MDO). Understanding these differences is essential for accurate diagnosis, surgical planning, and the prediction of outcomes in patients with PRS.

**Figure 1.**
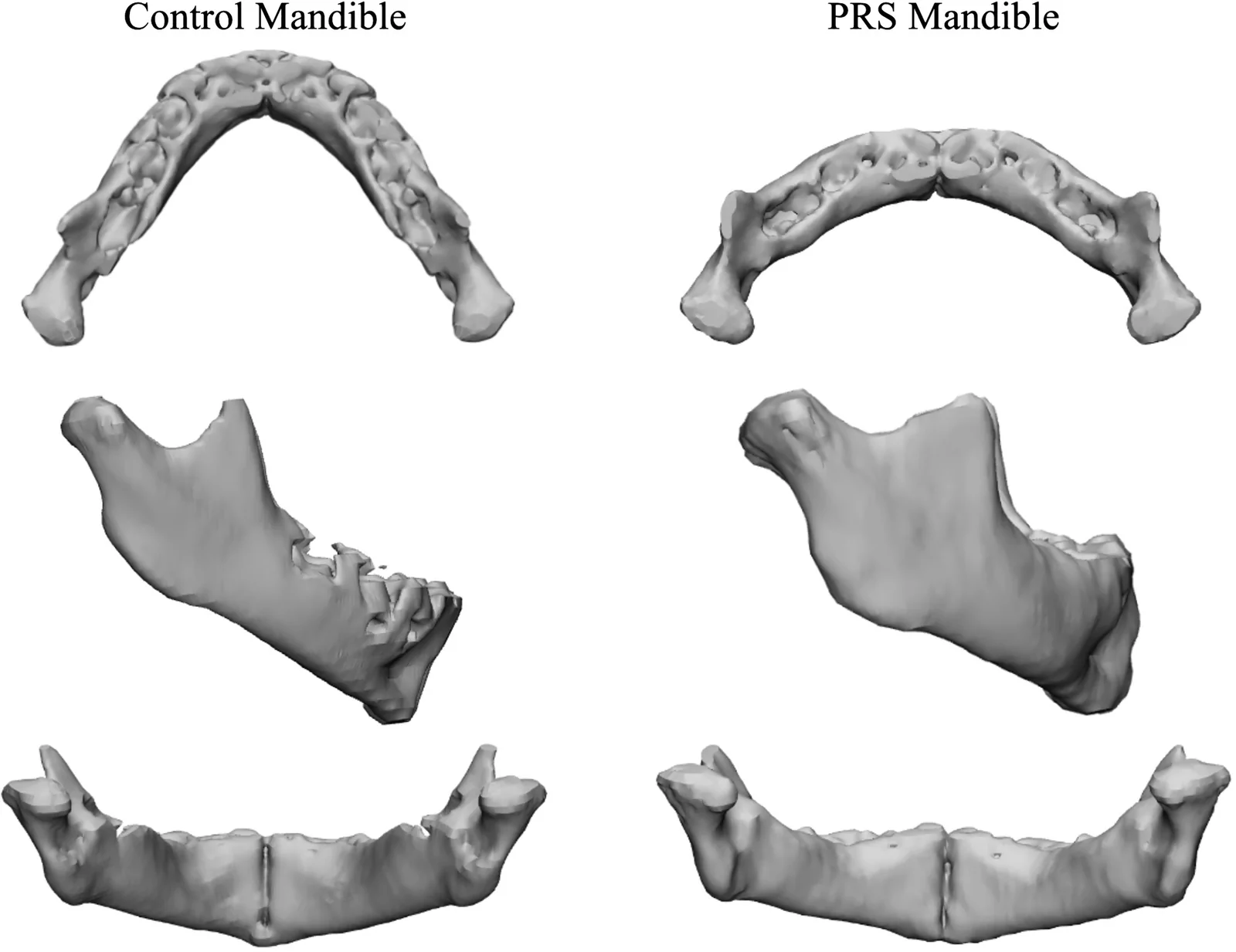
Shown left: mandible of patient in control group. Shown right: mandible of patient with PRS. From top to bottom: superior, lateral, and posterior views of a neonatal mandible.

Previous studies have largely focused on clinical observations and less precise methods of measurement, such as ultrasound, leading to inconsistencies in the literature regarding the extent and nature of mandibular abnormalities in PRS.^9–13^ These early diagnostic methods for mandible anomalies are highly relevant, allowing for sensitive detection and timely intervention for life-threatening airway obstruction. However, the advent of computed tomography (CT)-based imaging techniques such as 3D morphometrics now allows for highly detailed and accurate anatomic measurement of craniofacial structures. This is of concern in more severe cases of PRS where surgical intervention is oftentimes considered, which is the focus of this study.

This study aims to contribute to the literature by providing an extensive morphometric analysis of the mandibles in neonates with PRS compared to healthy controls. By identifying key differences in mandibular morphology, the authors hope to improve and better define the understanding of micrognathia's structural role in PRS and enhance the clinical management of this complex condition with objective data for defining micrognathia. Specifically, this study explores whether PRS mandibles exhibit patterns of asymmetry and dimensional differences that contribute to the characteristic clinical features of the sequence. The findings may aid in the development of future indications for MDO or the timing of referrals to a specialist for patients with suspected micrognathia.

## Methods

### Data Collection

A single center retrospective case-control study using prospectively collected data was conducted using patients with non-syndromic PRS or controls without any craniofacial anomalies presenting to our institution between 2011 and 2024. Twenty-five non-syndromic patients with PRS who received MDO were age-matched based on age at CT scan with twenty-five patients without PRS who met inclusion and exclusion criteria.

Inclusion criteria for patients with PRS included 1) diagnosis of non-syndromic PRS; 2) a CT scan of the head including the full mandible before any operative intervention occurred; 3) age less than 1 month at the time of CT scan; 4) must have later received MDO. Inclusion and exclusion criteria for controls included 1) no congenital or anatomical anomalies affecting the craniofacial structures; 2) a CT scan of the head including the full mandible; 3) the cause of CT scan should not relate to the fracture of any craniofacial structures; 4) age less than 1 month at time of CT scan.

The diagnosis of non-syndromic PRS was made clinically either prenatally or at birth, with a follow-up visit with a clinical geneticist to rule out associated syndromes. The age limit of less than 1 month was selected to focus on the neonatal period, which is a critical time for MDO related decision-making in patients with PRS.^14^ CT scans in the control group were typically obtained for patients requiring rapid evaluation of head trauma without fracture or for the workup of upper airway anomalies such as choanal atresia, piriform aperture stenosis, or laryngomalacia. CT was the chosen modality for this study as it provides the high-resolution bony detail essential for precise 3D morphometric analysis. All CT scans included in the study had a slice thickness of 1.25 mm or less to ensure adequate resolution for 3D reconstruction. CT scans were imported into Materialise Mimics 24.0 (Leuven, Belgium) to reconstruct 3D models of each skull for subsequent morphometric analysis in 3dMD Vultus (Atlanta, Georgia, USA). Demographics collected for each patient included age at CT scan, sex, and ethnicity.

### Mandible Morphometric Analysis

Based on previous morphometric studies, analysis included one angular and 12 linear measurements of the mandible.^15–19^ The initial identification of 11 landmarks was performed by two independent reviewers and corroborated by a pediatric neuroradiologist to reach a final consensus before any measurements were extracted. Linear measurements from 11 mandibular landmarks were calculated, and one ratio was calculated. The intercondylar angle was computed, and the linear measurements included: intercondylar distance, intergonial distance, body length, body width, body height, effective mandibular length, pogonion-interdental distance, condylar width, condylar height, ramus width, and ramus height. Measurements were calculated bilaterally as necessary and averaged for comparison when applicable (Figures 2 and 3). The ratio calculated was ramus height to body length.

**Figure 2.**
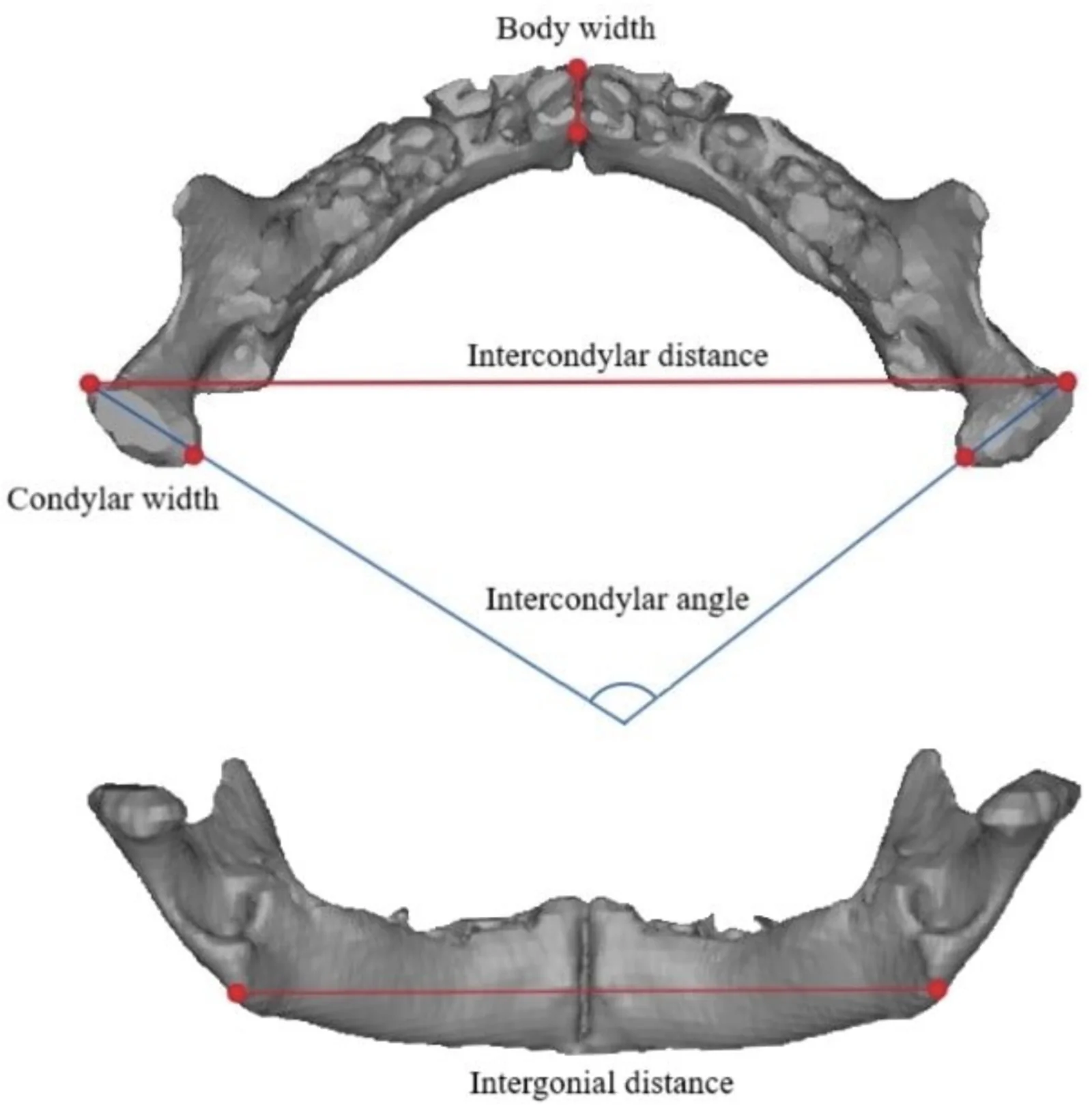
Superior and posterior views of a neonatal PRS mandible with anatomic depictions of intercondylar angle, intercondylar distance, body width, condylar width, and intergonial distance.

**Figure 3.**
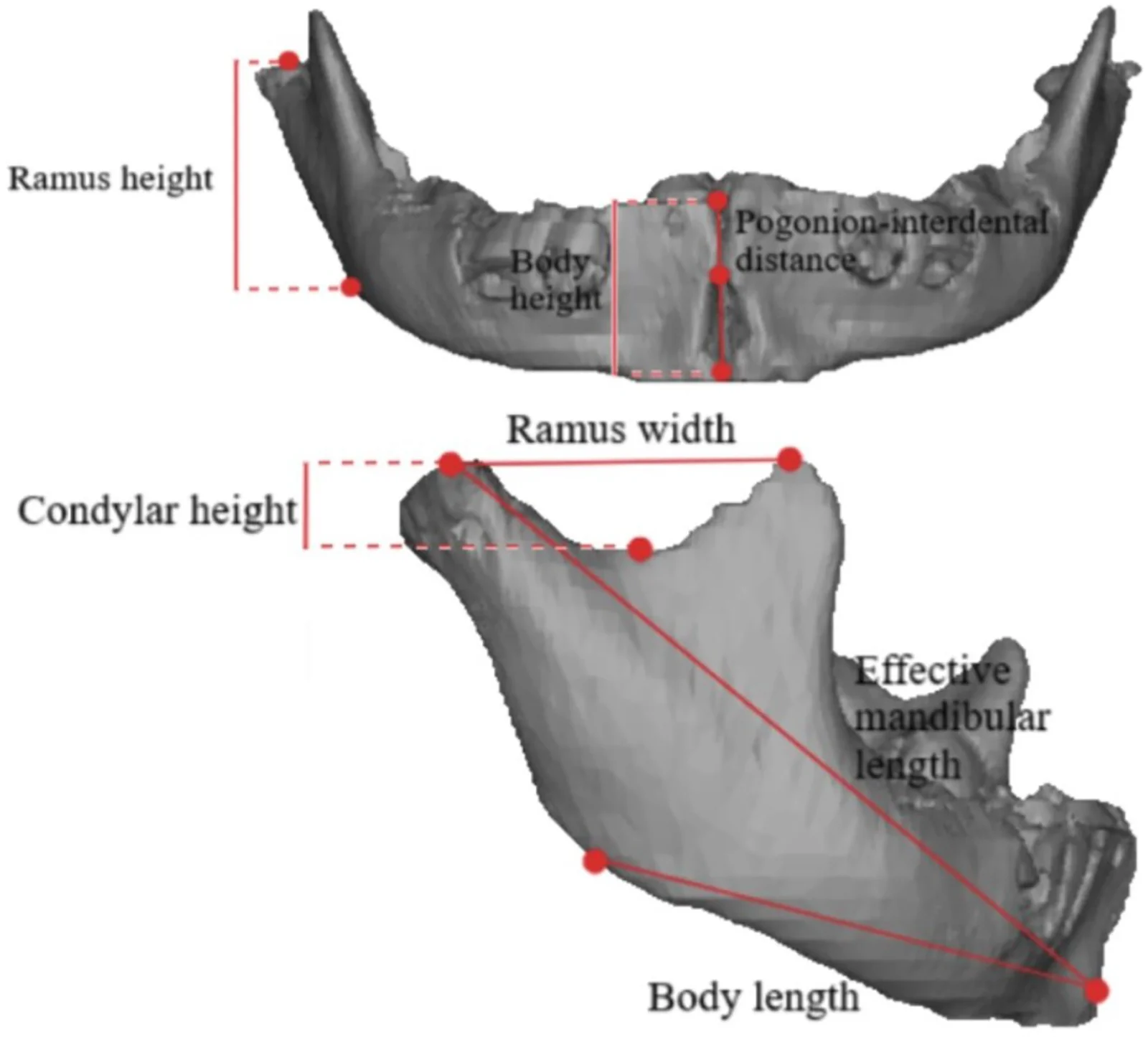
Anterior and lateral views of neonatal PRS mandible with anatomic depiction of body length, body height, pogonion-interdental distance, effective mandibular length, condylar height, ramus height, and ramus width.

For bilateral measurements, asymmetry indices were calculated using the following formula^20^:

$$Absolute\;asymmetry\;index=\left|\frac{Right\;Measurement-Left\;Measurement}{Right\;Measurement+Left\;Measurement}\right|\times 100\text{\%}$$

### Statistical Analysis

Fisher's exact tests and Chi-square tests assessed any potential age and sex distribution differences between the case and control groups. A two-tailed paired samples t-test or its non-parametric counterpart (ie, Wilcoxon signed-rank test) was used to analyze continuous variables. Shapiro-Wilk statistics were calculated to evaluate the normality of data. Intraclass correlation coefficients quantified interrater reliability and exceeded 0.85. All data were analyzed at a significance level of 0.05 using Statistical Product and Service Solutions (SPSS) Statistics 29 (Armonk, New York, USA).

## Results

### Patient Demographics

Twenty-five neonates with non-syndromic PRS and 25 control neonates without PRS were included. The mean age of patients with PRS was 15 days at the time of head CT scan, ranging from 1 day to 28 days, and the mean age for controls was 14 days at the time of head CT scan, ranging from 1 day to 29 days. The PRS group consisted of 13 males (52%) and 12 females (48%), and the control group consisted of 16 males (64%) and 9 females (36%). The racial composition of each group was similar (Table 1). Of neonates with PRS, all received MDO.

**Table 1.** Patient Demographics.

	PRS	Control	*p*-value
Number of subjects	25	25	
Age (days)			.697
Mean ± SD	15 ± 10.5	14 ± 9.0	
Sex			.390
Male	13 (52%)	16 (64%)	
Female	12 (48%)	9 (36%)	
Ethnicity			.258
Asian	1 (4%)	0 (0%)	
Black	2 (8%)	2 (8%)	
Hispanic	7 (28%)	9 (36%)	
White	15 (60%)	14 (56%)	

### Morphometric Asymmetry Analysis

The absolute asymmetry index (%) was computed for each bilateral measurement in both groups. Compared to control neonates, the mandibular body length was significantly more asymmetrical in patients with PRS with a calculated asymmetry index of 3.27 ± 2.04 compared to 1.95 ± 0.98 in control neonates (*p* = .006). The asymmetry indices of condylar width (PRS: 2.73 ± 2.13; control: 3.33 ± 3.57; *p* = .474), condylar height (PRS: 6.05 ± 5.11; control: 5.30 ± 3.49; *p* = .552), effective mandibular length (PRS: 1.66 ± 1.68; control: 1.20 ± 0.89; *p* = .234), ramus width (PRS: 2.46 ± 2.17 ; control: 3.82 ± 3.10; *p* = .077), and ramus height (PRS: 5.06 ± 3.16; control: 3.49 ± 2.48; *p* = .057) were not found to be significantly different between patients with PRS and controls (Table 2).

**Table 2.** Morphometric Asymmetry Analysis: Pierre Robin Sequence versus Control.

	Mean ± SD			
Mandibular measurements	PRS (*n* = 25)	Control (*n* = 25)	Δ (PRS - control)	*p*-value
dBL^a^	3.27 ± 2.04	1.95 ± 0.98	1.32	.**006**
dCW	2.73 ± 2.13	3.33 ± 3.57	−0.60	.474
dCH	6.05 ± 5.11	5.30 ± 3.49	−5.25	.552
dEML	1.66 ± 1.68	1.20 ± 0.89	0.46	.234
dRW	2.46 ± 2.17	3.82 ± 3.10	−1.36	.077
dRH	5.06 ± 3.16	3.49 ± 2.48	1.57	.057

BL, body length; CW, condylar width; CH, condylar height; EML, effective mandibular length; RW, ramus width; RH, ramus height.

a Prefix ‘d’ indicates the absolute asymmetry index (%) for each measurement. Bold indicates statistical significance.

### Linear Morphometric Analysis

Linear morphometrics (millimeters) were computed for neonates with PRS and control neonates. PRS measurements significantly larger than control measurements included intercondylar distance (PRS: 65.73 ± 4.16; control: 63.47 ± 3.52; *p* = .044), intergonial distance (PRS: 52.90 ± 3.91; control: 49.99 ± 3.55; *p* = .008), pogonion-interdental distance (PRS: 10.60 ± 2.52; control: 9.05 ± 1.78; *p* = .015), ramus height (PRS: 18.48 ± 1.87; control: 17.28 ± 1.66; *p* = .001), and ramus height to body length ratio (PRS: 0.52 ± 0.08; control: 0.43 ± 0.05; *p* < .001). PRS measurements significantly smaller than control measurements included intercondylar angle (PRS: 118.32 ± 7.15; control: 124.31 ± 3.24; *p* < .001), body length (PRS: 35.50 ± 3.16; control: 40.15 ± 2.65; *p* < .001), and effective mandibular length (PRS: 47.42 ± 3.13; control: 51.71 ± 3.15; *p* < .001) (Table 3).

**Table 3.** Linear Morphometric Analysis: Pierre Robin Sequence versus Control Measurements (millimeter).

	Mean ± SD			
Measurement	PRS (*n* = 25)	Control (*n* = 25)	Δ (PRS - control)	*p*-value
Intercondylar angle^a^	118.32 ± 7.15	124.31 ± 3.24	−5.99	**<**.**001**
Intercondylar distance	65.73 ± 4.16	63.47 ± 3.52	2.26	.**044**
Intergonial distance	52.90 ± 3.91	49.99 ± 3.55	2.91	.**008**
Body length	35.50 ± 3.16	40.15 ± 2.65	−4.65	**<**.**001**
Body width	6.26 ± 0.77	6.71 ± 0.92	−0.45	.070
Body height	13.23 ± 1.66	12.77 ± 1.70	0.46	.340
Effective mandibular length	47.42 ± 3.13	51.71 ± 3.15	−4.29	**<**.**001**
Pogonion-interdental distance	10.60 ± 2.52	9.05 ± 1.78	1.55	.**015**
Condylar width	8.63 ± 1.19	8.63 ± 1.09	0.00	.992
Condylar height	9.26 ± 1.31	9.53 ± 1.29	−0.27	.304
Ramus width	14.81 ± 1.39	14.70 ± 1.55	0.11	.715
Ramus height	18.48 ± 1.87	17.28 ± 1.66	1.20	.**001**
Ramus height to body length ratio	0.52 ± 0.08	0.43 ± 0.05	0.09	**<**.**001**

Intercondylar angle, angle of the intersection of the mediolateral axis of each condyle; Intercondylar distance, lateral aspect of left condyle to lateral aspect of right condyle; Intergonial distance, left gonion to right gonion; Body length, gonion to pogonion; Body width, anterior–posterior distance of mandibular body from the infradentale; Body height, menton to infradentale, Effective mandibular length, condylion to pogonion; Pogonion-interdental distance, pogonion to infradentale; Condylar width, lateral to medial point of each condyle; Condylar height, condylion to mandibular notch; Ramus width, condylion to coronoid process; Ramus height, condylion to gonion.

a Measured in degrees. Bold indicates statistical significance.

Measurements (millimeters) that were not found to be significantly different between neonates with PRS and control neonates included body width (PRS: 6.26 ± 0.77; control: 6.71 ± 0.92; *p* = .070), body height (PRS: 13.23 ± 1.66; control: 12.77 ± 1.70; *p* = .340), condylar width (PRS: 8.63 ± 1.19; control: 8.63 ± 1.09; *p* = .992), condylar height (PRS: 9.26 ± 1.31; control: 9.53 ± 1.29; *p* = .304), and ramus width (PRS: 14.81 ± 1.39; control: 14.70 ± 1.55; *p* = .715) (Table 3).

## Discussion

This study compared the morphometric data of the youngest and largest cohort of neonates with non-syndromic PRS with age-matched controls to better understand the mandibular anatomic differences in PRS. The overall findings indicate that the micrognathia observed in neonates with PRS is constituted by a wide and antero-posteriorly short mandible for its height. Asymmetry is also common in condyles and rami of healthy mandibles. Except for body length, the mandibles of neonates with PRS were observed to be similarly asymmetric compared to controls.

The clinical significance of increased asymmetry in mandibular body length warrants further investigation. While differential distraction lengths and/or vectors on either side may be considered to achieve postoperative symmetry, the current literature does not establish pre-operative mandibular asymmetry as a predictive factor for surgical outcomes or post-distraction growth patterns in patients with isolated PRS.^5,7,16^ The findings of this study may help inform surgical planning for MDO. For instance, the finding of a significantly shorter mandibular body but a relatively preserved ramus height suggests that a distraction vector primarily targeting antero-posterior lengthening of the body may be effective in this population. This is particularly relevant to discussions regarding the various distraction vectors, as some studies suggest a vertical component to the deformity.^7,8,21^ However, our findings of a modestly increased ramus height in this neonatal cohort (*p* = .001) indicate that the primary deficiency is antero-posterior, reinforcing that a surgical plan focused on the sagittal vector is the most deficient vector to correct. Furthermore, the characteristic features of PRS mandibles such as wider intergonial and intercondylar distances should be noted in this population when considering surgical distraction design to achieve normal mandibular anatomy.

The morphometric data on neonatal mandibular anatomy in this study could inform future research into objective assessment tools for assessing micrognathia. Currently, the decision to perform MDO is primarily based on the severity of airway obstruction as assessed by clinical examination, polysomnography, and endoscopic evaluation, rather than on mandibular morphology alone. Although CT imaging is routinely obtained in infants with Robin sequence being evaluated prior to undergoing MDO to assess mandibular anatomy and airway configuration, the role of specific morphometric thresholds in guiding treatment decisions is undefined. One previous study identified CT-based measures of tongue position and mandibular configuration that differentiate infants requiring tracheotomy from those managed less invasively.^22^ While this work suggests that imaging of tongue posture may help identify patients at higher risk of persistent obstruction, CT inherently captures only a static snapshot of a dynamic soft-tissue structure. Tongue position on CT can be influenced by sedation, cooperation, and respiratory phase, and the reproducibility of such measurements remains uncertain. Accordingly, we view CT-based tongue assessment, along with other objective imaging parameters, as a supportive tool that may serve as a complementary investigation to clinical assessment.

On evaluation of morphometric studies of PRS, one study reported a smaller ramal height in patients with PRS (16.7 mm) compared to controls (17.3 mm), but the finding was not statistically significant (*p* = .346).^16^ The present study found the ramal height to be significantly larger in patients with PRS (18.48 mm) compared to controls (17.28 mm). Other studies have also identified a globally hypoplastic mandible in infants with PRS.^21^ Zellner et al. reported a more obtuse anterior symphyseal angle, which corroborates our findings of an increased intergonial distance, suggesting a splaying of the mandibular bodies compared to controls. However, a notable difference exists in the measurement of ramus height. Zellner et al. observed a significantly shorter ramus in infants with PRS, while the present study found a modest increase in ramus height. This difference may be attributed to several factors, including a younger average age in our cohort (15 days versus 37 days), and the exclusion of patients with syndromic PRS.

Other studies evaluating mandibles in infants with PRS and without PRS found that PRS mandibles are shorter in length and display larger intergonial distances, consistent with the widening seen in the present study with a PRS intergonial distance of 52.90 mm ± 3.91 compared to the control measurements of 49.99 mm ± 3.55 (*p* = .008).^16,23^ This widening is accompanied by a smaller intercondylar angle and reduced body length, which reflect constricted bounds of the oral cavity in the anterior-posterior direction. Albeit these studies were performed with comparatively older aged cohorts of pediatric patients, which cannot be generalized to patients in the immediate postnatal period as in the present study. Additionally, not all these studies collected data using high fidelity 3D reconstructions.

The restricted oral cavity space created by mandibular anatomic differences may subsequently cause glossoptosis, an integral finding in PRS.^24^ In PRS, the narrower oral cavity restricts forward tongue positioning, thus facilitating the tongue to rest posteriorly towards the oropharynx. Additionally, the smaller intercondylar angle represents a narrower mandible that reduces lateral space within the oral cavity, further restricting tongue mobility and promoting posterior displacement.

The finding of wider mandibles in neonates with PRS requires further consideration, and this may be a compensatory change. The primary defect of a shortened mandible often leads to glossoptosis. This displaced tongue posture may exert lateral force on the developing mandibular arches, which may promote a wider, U-shaped arch rather than the typical V-shape. This structural compensatory change could exacerbate the antero-posterior deficiency of the airway in neonates with PRS.

Clinically, glossoptosis creates airway obstruction and can necessitate interventions such as MDO.^1,21,25^ MDO directly lengthens the body of the mandible and relieves airway obstruction by enlarging the oral cavity space.^21,23,25^ By addressing the antero-posterior deficiency, MDO allows the tongue to rest in the oral cavity away from the airway, remedying the structural limitations of the mandible identified in this study.

This study contained several limitations. The patient cohort in this study is inherently biased toward more severe presentations of PRS that required MDO. Therefore, our findings are not generalizable to the full spectrum of micrognathia found in PRS, especially milder cases which do not receive operative management. This investigation did not correlate other clinical data such as polysomnography results with the specific morphometric findings; such an analysis could help elucidate why anatomic findings do not always align with the severity of airway obstruction.^6,26^ While the cohort size in this study is among the largest in the literature, larger cohort studies among multiple institutions could enhance statistical power and confirm these observations across diverse populations, especially in the context of contrasting findings. Additionally, it is standard protocol to minimize radiation time when conducting CT scans at our institution. Consequently, all CT scans were not standardized in dose, which is improved with longer scan times. During the process of 3D reconstruction, lower dose scans may not have yielded reconstructions identical to the skull of the patient compared to patients with higher dose scans. This compromise can result in slight deviations in the reconstructed dimensions, especially in smaller anatomical structures such as the condyles.

## Conclusions

The findings indicate that severe micrognathia is constituted by a wide and antero-posteriorly short mandible for its height. Except body length, the mandibles of neonates with PRS are similarly asymmetrical compared to controls. The results may explain how mandibular structural differences reduce oral cavity space and promote glossoptosis and airway obstruction. Moreover, the measurements resulting from high fidelity 3D mandible reconstructions facilitate the establishment of normative mandibular data for control and neonates with PRS for potential use in future objectivization of the morphometric definitions of micrognathia and its role in directing operative management.
